# TRPV4 and K_Ca_ ion channels functionally couple as osmosensors in the paraventricular nucleus

**DOI:** 10.1111/bph.13023

**Published:** 2015-01-23

**Authors:** C H Feetham, N Nunn, R Lewis, C Dart, R Barrett-Jolley

**Affiliations:** 1 Institute of Ageing and Chronic Disease, Faculty of Health & Life Sciences, University of LiverpoolLiverpool, L69 3GA, UK; 2 Faculty of Life Sciences, University of ManchesterManchester, M13 9PT, UK

## Abstract

**Background and Purpose:**

Transient receptor potential vanilloid type 4 (TRPV4) and calcium-activated potassium channels (K_Ca_) mediate osmosensing in many tissues. Both TRPV4 and K_Ca_ channels are found in the paraventricular nucleus (PVN) of the hypothalamus, an area critical for sympathetic control of cardiovascular and renal function. Here, we have investigated whether TRPV4 channels functionally couple to K_Ca_ channels to mediate osmosensing in PVN parvocellular neurones and have characterized, pharmacologically, the subtype of K_Ca_ channel involved.

**Experimental Approach:**

We investigated osmosensing roles for TRPV4 and K_Ca_ channels in parvocellular PVN neurones using cell-attached and whole-cell electrophysiology in mouse brain slices and rat isolated PVN neurons. Intracellular Ca^2+^ was recorded using Fura-2AM. The system was modelled in the NEURON simulation environment.

**Key Results:**

Hypotonic saline reduced action current frequency in hypothalamic slices; a response mimicked by TRPV4 channel agonists 4αPDD (1 μM) and GSK1016790A (100 nM), and blocked by inhibitors of either TRPV4 channels (RN1734 (5 μM) and HC067047 (300 nM) or the low-conductance calcium-activated potassium (SK) channel (UCL-1684 30 nM); iberiotoxin and TRAM-34 had no effect. Our model was compatible with coupling between TRPV4 and K_Ca_ channels, predicting the presence of positive and negative feedback loops. These predictions were verified using isolated PVN neurons. Both hypotonic challenge and 4αPDD increased intracellular Ca^2+^ and UCL-1684 reduced the action of hypotonic challenge.

**Conclusions and Implications:**

There was functional coupling between TRPV4 and SK channels in parvocellular neurones. This mechanism contributes to osmosensing in the PVN and may provide a novel pharmacological target for the cardiovascular or renal systems.

## Tables of Links

**Table:** 

TARGETS
**Ion channels**
TRPV4 channel
SK channels, KCa2.x
BK channel, KCa1.1
KCa channels
IK channel, IKCa1, KCa3.1

**Table:** 

LIGANDS
4αPDD
GSK1016790A
HC067047
IbTX, iberiotoxin
RN1734
TRAM-34
UCL-1684

These Tables list key protein targets and ligands in this article which are hyperlinked to corresponding entries in http://www.guidetopharmacology.org, the common portal for data from the IUPHAR/BPS Guide to PHARMACOLOGY (Pawson *et al*., 2014) and are permanently archived in the Concise Guide to PHARMACOLOGY 2013/14 (Alexander *et al*., 2013).

## Introduction

The transient receptor potential channel 4 (TRPV4) is a well-established mechanosensitive ion channel whose function is important for cell volume regulation (Becker *et al*., 2005; Phan *et al*., 2009; Guilak *et al*., 2010; Benfenati *et al*., 2011). Frequently, cellular responses to changes in osmolality involve TRPV4-mediated elevation of intracellular Ca^2+^ (Liedtke *et al*., 2003b) and consequent activation of calcium-activated potassium channels (K_Ca_) (Strotmann *et al*., 2003; Gao and Wang, 2010; Mobasheri *et al*., 2010; Sonkusare *et al*., 2012). Genetic deletion of TRPV4 channels results in blunted autonomic response to osmotic challenge (Liedtke and Friedman, 2003a) and the hypothalamus is likely to be involved as it is a key centre for control of the autonomic nervous system and richly expresses both TRPV4 (Guler *et al*., 2002) and K_Ca_ channels (Sausbier *et al*., 2006; Gui *et al*., 2012). There are several subtypes of calcium-activated potassium channels, namely K_Ca_2.1 (SK or SK1), K_Ca_2.2 (SK or SK2), K_Ca_2.3 (SK or SK3), K_Ca_3.1 (SK4, or simply IK) and Slo (BK). Activation of one or more of these channels will result in reduced membrane excitability and hyperpolarization. It has also been suggested that membrane hyperpolarization will increase the driving force for Ca^2+^ entry, leading to an increase in intracellular Ca^2+^, activating further K_Ca_ channels in a positive feedback loop (Watanabe *et al*., 2002). Such hyperpolarization of autonomic neurones would be expected to have widespread physiological consequences, such as a decrease in BP or sympathetic activity due to decreased firing rate.

Body fluid osmolality is usually regulated within a narrow range (∼290–300 mOsm) and previous studies show that application of hypertonic saline to the hypothalamus increases BP (Chen and Toney, 2001; Bourque, 2008; Chu *et al*., 2010), whereas hypotonic challenge decreases sympathetic nerve activity, BP and heart rate (Bourque and Oliet, 1997). At least some of this activity appears to be mediated by the paraventricular nucleus (PVN) of the hypothalamus (Holbein *et al*., 2014). Furthermore, water deprivation increases expression of the early response gene c-*fos* in pre-autonomic parvocellular neurones of the PVN (Stocker *et al*., 2004), a nucleus with an established role in both osmotic homeostasis (Bourque, 2008) and central cardiovascular control (Nunn *et al*., 2011).

In the current work, we tested the hypothesis that hypotonicity decreased action potential frequency in parvocellular neurones by activating TRPV4 channels, which increases intracellular Ca^2+^ sufficiently to hyperpolarize neurones via secondary activation of K_Ca_ channels. We characterized ion channel specificity using patch-clamp electrophysiology in hypothalamic slices. We used these experimental data in an *in silico* model of the system to predict whether our proposed hypothesis was possible. Finally, we measured intracellular Ca^2+^ responses of isolated neurones to verify this experimentally. Overall, we found that osmolality regulated PVN neurones through a mechanism involving TRPV4 and SK ion channels.

## Methods

### Animals

All animal care and experimental procedures complied with the regulations of the Home Office, UK and were approved by the Ethical Review Committee of the University of Liverpool. All studies involving animals are reported in accordance with the ARRIVE guidelines for reporting experiments involving animals (Kilkenny *et al*., 2010; McGrath *et al*., 2010). A total of 75 CD1 mice and 15 Wistar rats were used in the experiments described here. CD1 mice and pregnant Wistar rats for the use of pups, were purchased from Charles River (Kent, UK), and kept under standard 12 h/12 h light/dark conditions with unlimited access to water and normal chow diet.

### Brain slice preparation

CD1 mice of both sexes, aged 2–3 weeks were killed by Schedule 1 methods. The brain was swiftly removed and placed in an ice-cold, low Na^+^, high sucrose artificial cerebro-spinal fluid (ACSF composition (mM): 95 NaCl, 1.8 KCl, 1.2 KH_2_PO_4_, 0.5 CaCl_2_, 7 MgSO_4_, 26 NaHCO_3_, 5 glucose and 50 sucrose); then the hypothalamus was blocked and sliced as previously described (Barrett-Jolley *et al*., 2000). Coronal slices 300 μm thick were sliced using a Campden Instruments Ltd. 752 M Vibroslice (Leics, UK) and stored in a multi-well dish containing physiological ACSF kept at 35–37°C with continuous perfusion of 95% O_2_/5% CO_2_ and left to recover for at least 1 h before recording.

### Preparation of isolated neurones

Briefly, Wistar rats (aged 2–6 days old, of both sexes) were killed by Schedule 1 methods. Rats were used to ensure that the area isolated was indeed PVN tissue as the area is larger and could therefore be isolated with greater confidence. There are few known differences between the PVN of these two rodent species. The brain was removed and stored in ice-cold PBS, whereas the hypothalamus was blocked and sliced as previously described (Barrett-Jolley *et al*., 2000). Hypothalamic brain slices were cut to 600 μm on a Campden Instruments Ltd. 752 M Vibroslice and the PVN area was punched out using a fire-polished glass pipette. The area punched was 1 mm in diameter, located at the top of the third ventricle, as shown in Figure 8H. At the conclusion of isolated neurone work, we performed Western blot analysis against corticotropin-releasing factor (CRF), a typical marker of PVN neurones (Figure 8I). Tissue was then transferred to Neurobasal™ medium supplemented with 0.5 mM l-glutamine (Sigma-Aldrich, Dorset, UK), 2% penicillin/streptomycin and 1% amphotericin (Sigma-Aldrich) and triturated until the cells were dispersed. Neurones were washed twice with fresh media and grown in a monolayer culture with 10% fetal calf serum. Isolated PVN neurones were plated out on glass-bottomed dishes for measurement of calcium and onto glass coverslip shards for whole-cell patch-clamp electrophysiology.

All cell culture reagents were from Invitrogen, Paisley, UK, unless stated otherwise.

### Western blotting

Isolated cells from rat PVN punches were lysed in 200 μL of ice-cold lysis buffer (mM): 20 Tris–HCl, pH 8.0; 250 NaCl; 3 EDTA, 3 EGTA, 0.5% (v/v) Triton X-100 and containing 1% (v/v) protease inhibitor cocktail (Sigma-Aldrich). The homogenate was centrifuged at 16 100× *g* for 10 min at 4°C and the resultant supernatant was removed and heated at 98°C for 5 min in an equal volume of 2× Laemmli sample buffer (Sigma-Aldrich). Proteins within the sample were resolved by SDS-PAGE on 10% polyacrylamide–Tris gels and transferred electrophoretically onto nitrocellulose membranes (Hybond ECL, GE Healthcare, Buckinghamshire, UK). Immunoblotting was performed as previously described (Sampson *et al*., 2003). Blots were incubated in anti-CRF antibody (Abcam ab8901; Cambridge, UK) at a dilution of 1:2000 overnight at 4°C, followed by incubation with anti-rabbit IgG HRP-linked secondary antibody (Cell Signaling Technology, Leiden, The Netherlands) at a dilution of 1:2000.

### Action current electrophysiology recordings

Hypothalamic brain slices were used for the recording of action currents in cell-attached mode. Patch-clamp electrophysiology was performed using an Axopatch 200b amplifier (Axon Instruments, Sunnyvale, CA, USA). Analogue data were further amplified with a Tektronix FM122 (Beaverton, OR, USA) AC-coupled amplifier. Low-pass filtering was set to 1 kHz and data were digitized at 5 kHz with a Digidata 1200B interface. Neurones were visualized using a Hitachi KP-M3E/K CCD camera (Hitachi, Berkshire, UK) attached to a Nikon Eclipse microscope (Nikon, Surrey, UK) with an effective magnification of ∼1000×. Analysis was performed using WinEDR (Dr. John Dempster, University of Strathclyde). Data were normalized by action current frequency (ACf)/initial average ACf.

Thick-walled patch pipettes were fabricated using fire-polished 1.5 mm o.d. borosilicate glass capillary tubes (Sutter Instrument, Novato, CA, USA, supplied by INTRACEL, Herts, UK). They were pulled using a two-step electrode puller (Narishige, Tokyo, Japan) and when filled had a resistance of approximately 8 MΩ.

Patched cells were chosen according to area and morphology. All cells were first checked for osmotic sensitivity by superfusing with hypotonic ACSF (about 270 mOsm; see later for composition) before addition of any drugs to the solutions. Only those that responded to the reduction in osmolality were then used for further analysis (10/15 cells responded to osmolality). Frequency analysis before treatments was performed 5 min before solutions were changed in order to obtain a steady baseline. Similarly, analysis during treatment was performed for 5 min once the solution had reached the recording chamber (2 min after solutions had been switched).

### Single-channel electrophysiology recordings

Recording setup for these measurements was the same as that used in action current recordings, with the exception of the use of the Tektronix FM122 AC coupled amplifier (see the section Action current electrophysiology recordings). Single-channel all-points amplitude histograms were created in WinEDR. Amplitudes were taken at various holding potentials and used to create current–voltage (*IV*) curves. Reversal potential (*V*_rev_) and slop conductance (*g*) were then calculated from the equation of the fitted line. Open probabilities and single channel kinetics were analysed using the QuB software (Qin *et al*., SUNY, Buffalo, NY, USA) following the methods of Lewis *et al*. (2013) (see also the Supporting Information).

### Design of the computer model

We constructed a NEURON (Hines and Carnevale, 1997) model of parvocellular neurones. Parameters come both from earlier published results and our current experiments where possible. Inputs arise from both excitatory ‘Netstim’ neurones and inhibitory interneurones. The interneurones are also driven by excitatory ‘Netstim’ neurones, based upon our previous model of spinally projecting parvocellular autonomic neurones (Lewis *et al*., 2010). To this model, we have added a general model for the K_Ca_ channel modified from Moczydlowski and Latorre (1983) and TRPV4 channel conductance taken from Deng *et al*. (2010), with a permeability ratio 1:1:6 for Na^+^, K^+^ and Ca^2+^, respectively, using the algorithm of Strotmann *et al*. (2003) (Figure 5A):

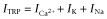
1

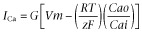
2

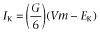
3

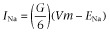
4

where *I* is the whole-cell current, *z* is the valency, *Cao* is the extracellular Ca^2+^ concentration, *Cai* is the intracellular Ca^2+^ concentration, *Vm* is the membrane potential and *E* is the reversal potential. *R*, *T* and *F* have the standard definitions.

We were able to alter osmolality within the model; hypo-osmolality is then assumed to activate TRPV4 channels in a sigmoidal manner:

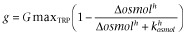
5

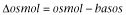
6where *g* is the conductance of the channel, *osmol* is the current osmolality, *basos* is the base osmolality (lowest used in our experiments is 270 mOsm) and *h* is the slope of activation by osmolality. For model parameters, see Table 1.

**Table 1:** TRPV4 channel parameters used in the NEURON simulation

Parameter		Unit	Meaning	Source
*Gmax*_TRP_	0.35 × 10^−5^	S·cm^−2^	Maximum conductance for TRP channel	Deng *et al*. (2010)
*k*	1.5	mOsm	Dissociation constant	
*osmol*	320	mOsm	Current osmolality of solution	Dunn *et al*. (1973)
*basos*	280	mOsm	Base osmolality	Value used in our experiments
*k*1	9.4	nM·s^−1^	Forward rate	Estimated from fit of Ca^2+^ data (Figure 5)
*k*2	0.05	nM·s^−1^	Reverse rate	

The model simulates intracellular Ca^2+^ accumulation according to Carnevale and Hines (2006). Ca^2+^ equilibrium is maintained by the incorporation of a simple Ca^2+^ pump (Carnevale and Hines, 2006) and includes rate constants for both activation and inhibition of TRPV4 channels by Ca^2+^ (see Table 1). Elevated intracellular Ca^2+^ levels increase open probability of TRPV4 channels via a Ca^2+^/calmodulin activation complex, and further increasing levels inhibit the channel by a so-far unknown mechanism (Watanabe *et al*., 2002).

The model also includes sub-routines to simulate pharmacological block of TRPV4 and/or K_Ca_ channels via altering permeabilities of the channels.

### Whole-cell membrane potentials

Isolated PVN neurones were used for whole-cell patch-clamp electrophysiology recordings in current clamp. Recording setup for these measurements was the same as that used in action current recordings, with the exception of the use of the Tektronix FM122 AC-coupled amplifier (see the section Action current electrophysiology recordings).

Thick-walled patch pipettes were pulled using a two-step electrode puller (Narishige) and when filled had a resistance of approximately 5 MΩ.

### Measurement of calcium

Cells were pre-incubated with Fura-2AM (5 μM) (Invitrogen) for a minimum of 30 min at 37°C. The cells were then washed with HEPES ACSF (see later for composition) for around 15 min to allow complete de-esterization of the dye.

Fluorescence images were captured and analysed using an imaging system consisting of an inverted phase contrast microscope (Nikon Diaphot 300) with a ×40 Fluor Nikon objective, a high-speed wavelength switcher (Cairn Research Dual Optoled power supply), a PC-controlled Exi Aqua digital CCD camera (Q Imaging, Surrey, BC, Canada) and Micro-Manager 1.4 software (University of California, San Francisco, CA, USA). Regions of interest were highlighted and Fura-2AM was excited alternately at 355 and 380 nm, with fluorescence measured at intervals of 2 s. Intracellular Ca^2+^ concentration was calculated using Equation 7 [below, taken from Grynkiewicz *et al*. (1985)], after calibration using the calcium ionophore A23187 (1 μM) and EGTA (5 mM) (Sigma-Aldrich):

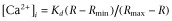
7where *K_d_* is the dissociation constant (taken as 145 nM at room temperature) for the Fura-2AM calcium binding, *R* is the ratio, and *R*_max_ and *R*_min_ are the ratio values measured under conditions of saturating calcium levels (A23187) and in the absence of calcium (EGTA) respectively.

Analysis was performed on measurements recorded before switching solutions to achieve a steady baseline. In addition, 1 min was allowed for the solution to reach the recording chamber and for solution exchange in this setup, and the peak of the response was taken over a period of 1 min for those measurements taken during treatment.

### Solutions

Low Na^+^, high sucrose ACSF composition (mM): 95 NaCl, 1.8 KCl, 1.2 KH_2_PO_4_, 0.5 CaCl_2_, 7 MgSO_4_, 26 NaHCO_3_, 5 glucose and 50 sucrose. Cell-attached patch recordings were made using the following solutions: ACSF composition (mM): 127 NaCl, 1.8 KCl, 1.2 KH_2_PO_4_, 2.4 CaCl_2_, 1.3 MgSO_4_, 26 NaHCO_3_ and 5 glucose. For low osmolality, ACSF (∼270 mOsmol) solutions were as normal ACSF above with 10 mM NaCl removed and osmolality was increased by the addition of sucrose for isotonic ACSF (300 mOsm). Solutions were bubbled with 95% O_2_/5% CO_2_ giving a pH of 7.4. Osmolality of solutions was measured with a freezing point Advanced Micro-Osmometer Model 3MO plus (Advanced Instruments Inc., Norwood, MA, USA). Pipette solution for action current recordings composition (mM): 35 KG; 5 KCl; 100 NaCl and 10 HEPES (pH 7.4) with NaOH (adjusted to 300 mOsm by the addition of sucrose). Pipette solution for single channel recordings composition (mM): 35 KG, 5 KCl, 100 NaMeSO_4_ and 10 HEPES (pH 7.4) with NaOH (adjusted to 300 mOsm with the addition of sucrose). Whole-cell recordings were made using the following solutions and recordings were adjusted for liquid junction potential. Extracellular ACSF composition (mM): 142 NaCl, 1.8 KCl, 1.2 KH_2_PO_4_, 2.4 CaCl_2_, 1.3 MgSO_4_, 5 glucose and 10 HEPES (pH 7.4) with NaOH (adjusted to 300 mOsm by the addition of sucrose). Intracellular pipette solution composition (mM): 115 KG, 26 KCl, 1 MgCl_2_, 5 EGTA and 10 HEPES (pH 7.2) with KOH (adjusted to 300 mOsm by the addition of sucrose). All experiments were performed in the daytime (11:00–17:00 h) to limit the effects of circadian rhythm on activity of the cells used (Belle *et al*., 2009).

### Data analysis

All data on graphs are shown as mean ± SEM. In all cases, ‘*n*’ refers to the number of experiments. In the case of dissociated neurone work, ‘*n*’ corresponds simply to the number of neurones. In the slice work, no more than one neurone was recorded per slice or animal and so ‘*n*’ refers to animals, slices and neurones. Simple comparisons were made using a one-tailed Student's paired *t*-test. Multiple comparisons were made using a general linear model with multiple comparisons by Tukey's *post hoc* test or against control levels using Dunnett's *post hoc* test (Minitab, Coventry, UK) where appropriate. A value of *P* < 0.05was taken as significant.

### Materials

Calcium ionophore A23187 (1 μM), 4-α-phorbol 12,13-didecanoate (4αPDD) (1 μM), GSK1016790A (100 nM), RN1734 (5 μM), HC067047 (300 nM), UCL-1684 (30 nM), TRAM-34 (30 nM) and iberiotoxin (IbTX) (1, 10, 30 and 100 nM) were all dissolved in DMSO and diluted to a final working concentration of no more than 0.01% DMSO (0.01% DMSO had no effect alone). Calcium ionophore A23187, TRPV4 channel agonist 4αPDD (Vincent *et al*., 2009), TRPV4 channel agonist GSK1016790A (Thorneloe *et al*., 2008) and BK channel inhibitor IbTX (Candia *et al*., 1992) were sourced from Sigma-Aldrich, and TRPV4 channel antagonist RN1734 (Vincent *et al*., 2009), TRPV4 channel antagonist HC067047 (Everaerts *et al*., 2010), SK channel inhibitor UCL-1684 and IK channel inhibitor TRAM-34 (Nehme *et al*., 2012) were sourced from Tocris, Bristol, UK.

## Results

### ACf changes with hypotonic solutions

**Figure 1:**
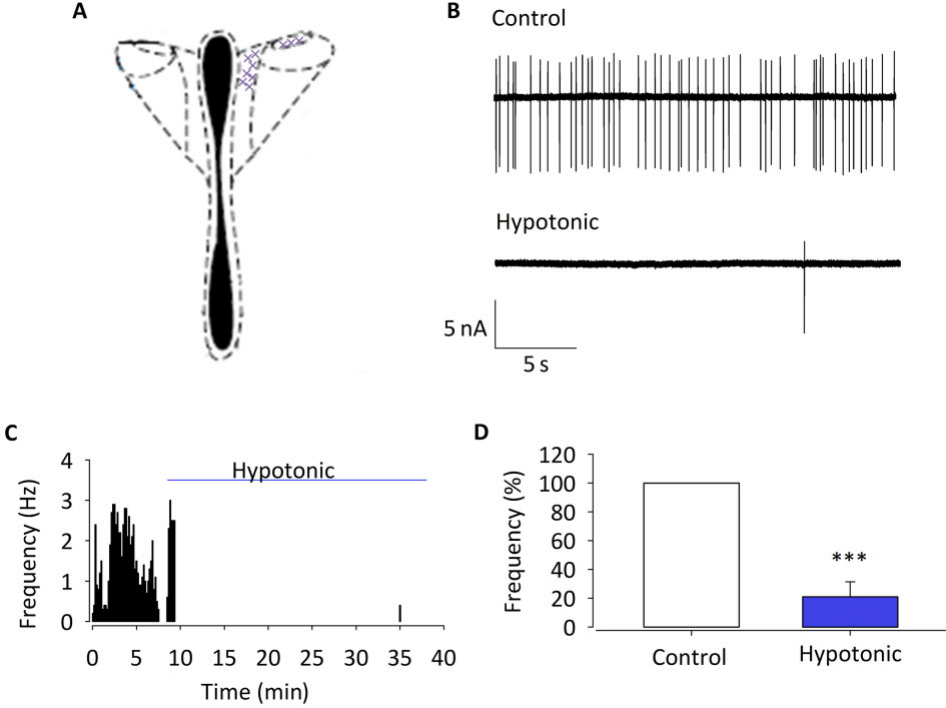
The effect of osmolality changes on ACf. Cell-attached action current measurements in slice recorded from location shown in (A). (B) Raw action current trace at 300 mOsm (control) and 270 mOsm (hypotonic challenge). (C) Representative frequency histogram showing action current response to hypotonic challenge of a single parvocellular neurone. (D) Percent normalized ACf forexperiments similar to those illustrated in (A) and (B) (normalized to ACf during control conditions) is significantly reduced. Data shown are means±SEM (*n*=10). ***P < 0.001, signficantly different from control.

To investigate the effect of osmolality changes on the activity of PVN neurones, ACf was recorded using cell-attached patch clamp. Brain slices were first equilibrated and superfused with isotonic ACSF (300 mOsm), which was then switched to hypotonic ACSF (∼280 mOsm). We recorded from anatomically and morphologically defined parvocellular neurones in the PVN (Figure 1A). ACf was significantly decreased upon hypotonic challenge with a 79 ± 10% reduction of ACf (Figure 1; *n* = 10; *P* < 0.001), confirming that the PVN has a capacity to detect and respond to changes in osmolality.

### ACf changes with TRPV4 channel agonists and antagonists

**Figure 2:**
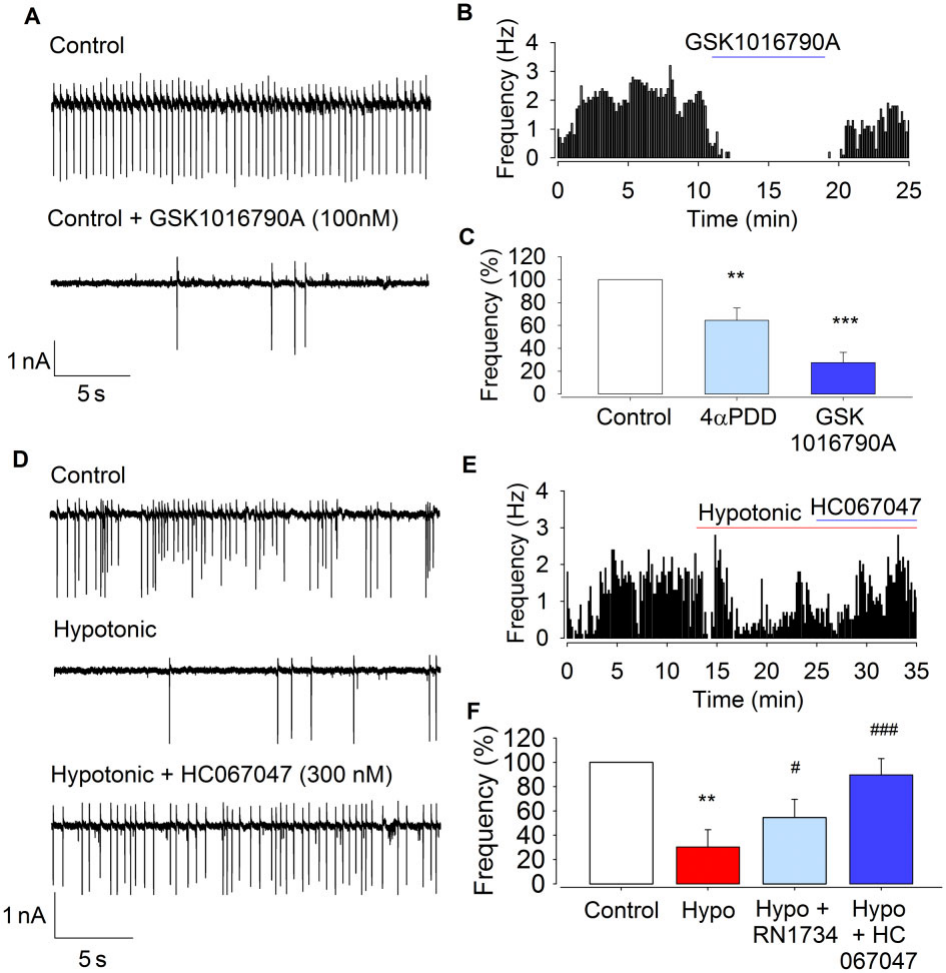
The effect of TRPV4 channel modulators on ACf. (A) Raw action current trace at 300 mOsm (control) with and without the selective agonist GSK1016790A (100 nM). (B) Representative frequency histogram showing action current responses to GSK1016790A. (C) ACf from experiments similar to those illustrated in (A) and (B) is significantly reduced upon addition of 4αPDD (*n* = 6) or GSK1016790A (*n* = 8). **P < 0.01, ***P < 0.001, signficantly different from control. (D) Raw action current trace at 300 mOsm (control), 270 mOsm (hypotonic) and with the addition of the TRPV4 channel antagonist HC067047 (300 nM). (E) Representative frequency histogram showing recovery of ACf upon HC067047 addition, after loss from hypotonic challenge. (F) Mean ACf from experiments similar to those illustrated in (D) and (E) is significantly reduced upon hypotonic challenge but not in the presence of RN1734 or HC101679A, both of which showed a significant difference to hypotonic alone. This further suggests a role for TRPV4 channels in osmosensation. **P < 0.01, signficantly different from control; #*P* < 0.05, ###*P* < 0.001, signficantly different from hypotonic alone; *n* = 6.

The TRPV4 channel is an established osmosensor (Mizuno *et al*., 2003) and has previously been shown to be expressed by parvocellular neurones (Carreno *et al*., 2009). Superfusion of parvocellular neurones with the selective TRPV4 channel agonist GSK1016790A (100 nM) reduced ACf by 72 ± 8%. 4αPDD (1 μM) also reduced ACf by 36 ± 10% (Figure 2A–C; *n* = 6; *P* < 0.05), although the effect seen with GSK1016790A was significantly greater (Figure 2C; *n* = 8; *P* < 0.01). Furthermore, the response to hypotonic solution was significantly reduced by the TRPV4 channel antagonists RN1734 (5 μM) and HC067047 (300 nM) (Figure 2D–F) compared with hypotonic alone: 70 ± 14% reduction versus hypotonic with RN1734: 45 ± 15% reduction (*n* = 6; *P* < 0.05), and a 10 ± 13% reduction with HC067047 in ACf (*n* = 6; *P* < 0.01). Antagonists had no significant effect on ACf when applied alone with isotonic solution (data not shown; *n* = 6).

### Identification of TRPV4 channels by single-channel recordings

**Figure 3:**
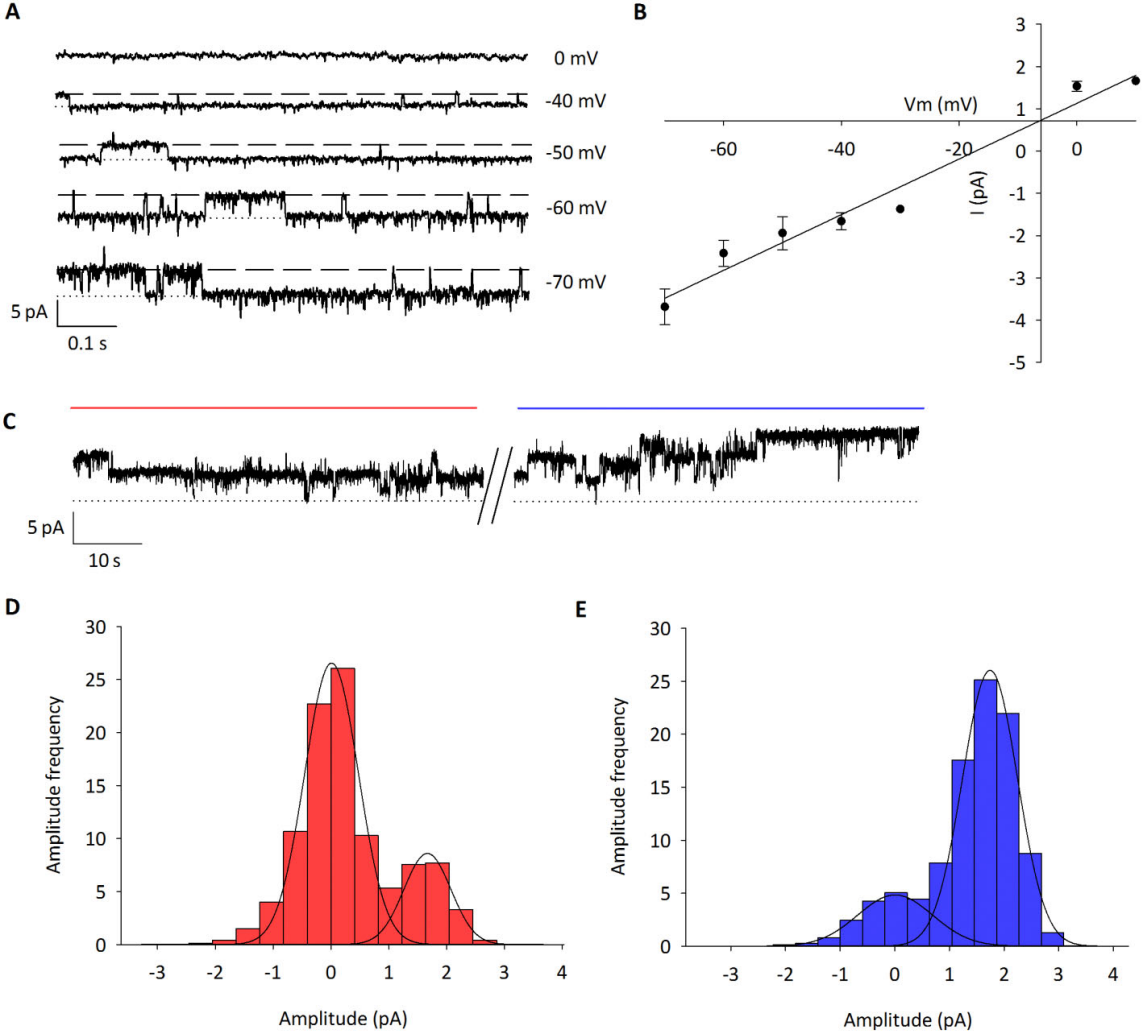
Single channel identification of the 4αPDD-activated channel. (A) Representative single-channel recordings in cell-attached patch clamp (dashed line represents open state). Channel activity was seen in 50% of patches (8/16). (B) *IV* curve shows mean slope unitary conductance of 57 ± 7 pS and reversal potential of −5 ± 3 mV (*n* = 6); indicative of a non-selective cation channel. (C) Representative single channel recordings before (red line) and after (blue line) 4αPDD at −40 mV. (D) Representative amplitude histogram before and (E) after addition of 4αPDD at −40 mV. *P*_0_ increased by 48 ± 9% (*n* = 4; *P* < 0.001) upon addition of 4αPDD (for kinetic analysis, see the Supporting Information).

To support the hypothesis that activation of TRPV4 channels was a direct action upon the parvocellular neurones, patched single-channel analysis was performed. Using voltage steps, a TRP-like channel was identified in 50% of recordings (8/16) with a conductance of 57 ± 7 pS and *V*_rev_ of −5 ± 3 mV (Figure 3A and B; *n* = 6; adjusted for junction potential). Furthermore, 4αPDD (1 μM) caused a significant increase in *P*_0_ of 48 ± 9% (Figure 3C–E; *n* = 4; *P* < 0.001), providing evidence that the channel identified in parvocellular neurones is TRPV4 (for additional kinetic analysis, see the Supporting Information).

### Pharmacological identification of K_Ca_ channels

**Figure 4:**
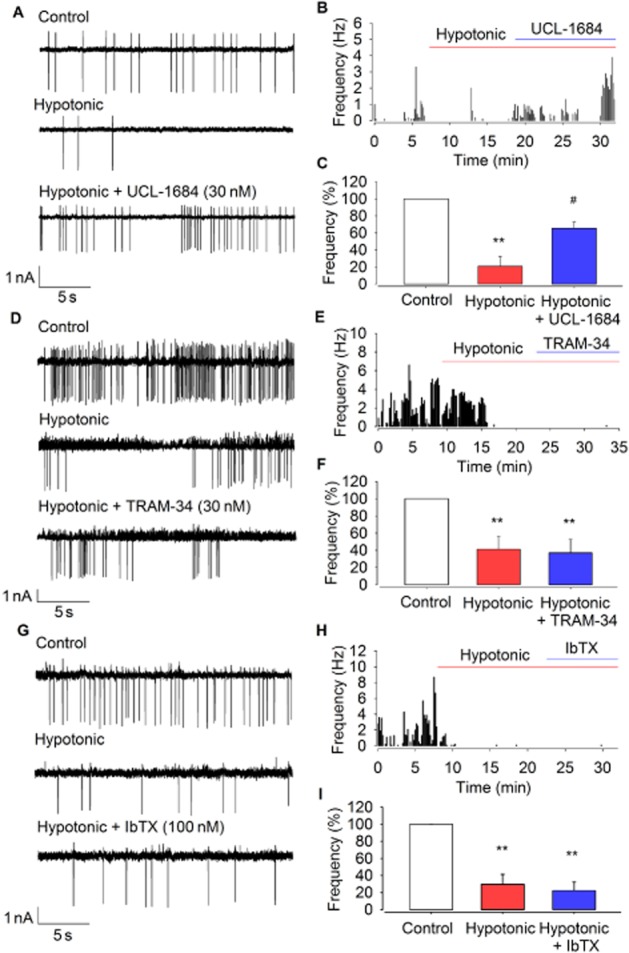
The effect of K_Ca_ channel inhibitors on osmotic sensitivity of PVN neurones. (A) Raw action current trace at 300 mOsm (control), 270 mOsm (hypotonic) and with the addition of the SK inhibitor UCL-1684 (30 nm). (B) Representative frequency histogram showing regain of ACf upon UCL-1684 addition after loss from hypotonic challenge. (C) Mean ACf from experiments similar to those illustrated in (A) and (B) is significantly reduced at 270 mOsm, but not in the presence of the SK channel inhibitor (*n* = 5). **P < 0.01, signficantly different from control; #*P* < 0.05, signficantly different from hypotonic alone. (D) Raw action current trace at 300 mOsm, 270 mOsm and with the addition of the IK channel inhibitor TRAM-34 (30 nm). (E) Representative frequency histogram showing TRAM-34 has no effect on ACf (F) Mean ACf from experiments similar to those illustrated in (D) and (E) is significantly reduced upon hypotonic challenge and this response is unchanged upon addition of TRAM-34 (*n* = 5). **P < 0.01, signficantly different from control. (G) Raw action current trace at 300 mOsm, 270 mOsm and with the addition of the BK channel inhibitor iberiotoxin (IbTX) (10 nm). (H) Representative frequency histogram showing IbTX has no effect on ACf. (I) Mean ACf from experiments similar to those illustrated in (G), and (H) is significantly reduced upon hypotonic challenge; osmotic sensitivity is unchanged upon addition of IbTX (*n* = 6). **P < 0.01, signficantly different from control.

Ca^2+^ entry via activation of TRPV4 channelswould be expected to activate K_Ca_ channels, and K_Ca_ channels have been identified in PVN neurones (Sausbier *et al*., 2006; Gui *et al*., 2012). We therefore investigated which of these K_Ca_ channels coupled hypotonicity to ACf. We found that the effects of hypotonic challenge on ACf were significantly reduced by the SK channel inhibitor UCL-1684 (30 nM) (Figure 4A–C; hypotonic alone: 79 ± 10% reduction in ACf vs. hypotonic with UCL-1684: 37 ± 7% reduction *n* = 5; *P* < 0.01). No significant changes in ACf were seen with UCL-1684 alone with isotonic solution (data not shown; *n* = 6). Osmotic sensitivity was not, however, significantly affected by the IK channel inhibitor TRAM-34 (Figure 4D–F; *n* = 5). The BK channel inhibitor IbTX was also without effect at concentrations of 1, 10, 30 or 100 nM (Figure 4G–I; *n* = 6). Together, these data suggest that only the SK channels were activated by TRPV4-mediated Ca^2+^ influx in these cells.

**Figure 5:**
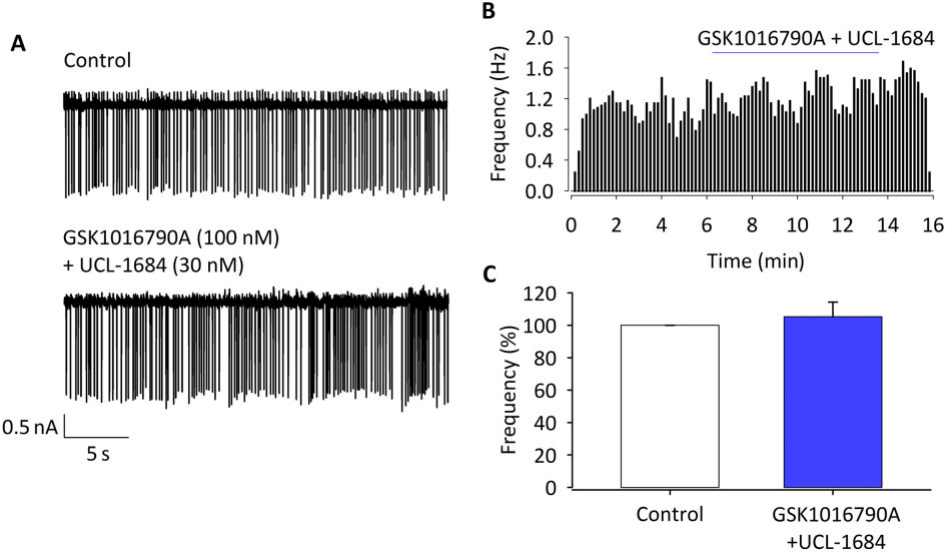
Simualtaneous activation of TRPV4 and inhibition of SK channels results in no change of action potential. (A) Raw ACf at 300 mOsm and with the addition of both GSK1016790A (100 nM) and UCL-1684 (30 nM). (B) Representative histogram showing that GSK1016790A and UCL-1684 together have no effect on ACf. (C) Mean ACf from experiments similar to those illustrated in (B) and (C) is not significantly changed with the addition of GSK1016790A and UCL-1684 (*n* = 7).

### Pharmacological evidence for coupling of TRPV4 and SK channels

If the reduction in ACf observed in response to TRPV4 channel activation results from a coupling between TRPV4 and SK channels, one would expect that an appropriate K^+^ channel blocker would prevent it. In order to identify a link between the TRPV4 and SK channels, ACf analysis was performed in slices with a combination of both the TRPV4 channel activator, GSK1016790A (100 nM) and the SK channel inhibitor, UCL-1684 (30 nM). With this combination of treatments, no significant difference in action current firing was observed (Figure 5A–C; *n* = 7; *P* > 0.05). These data provide support of our theory that these two channels are functionally linked.

### NEURON model

**Figure 6:**
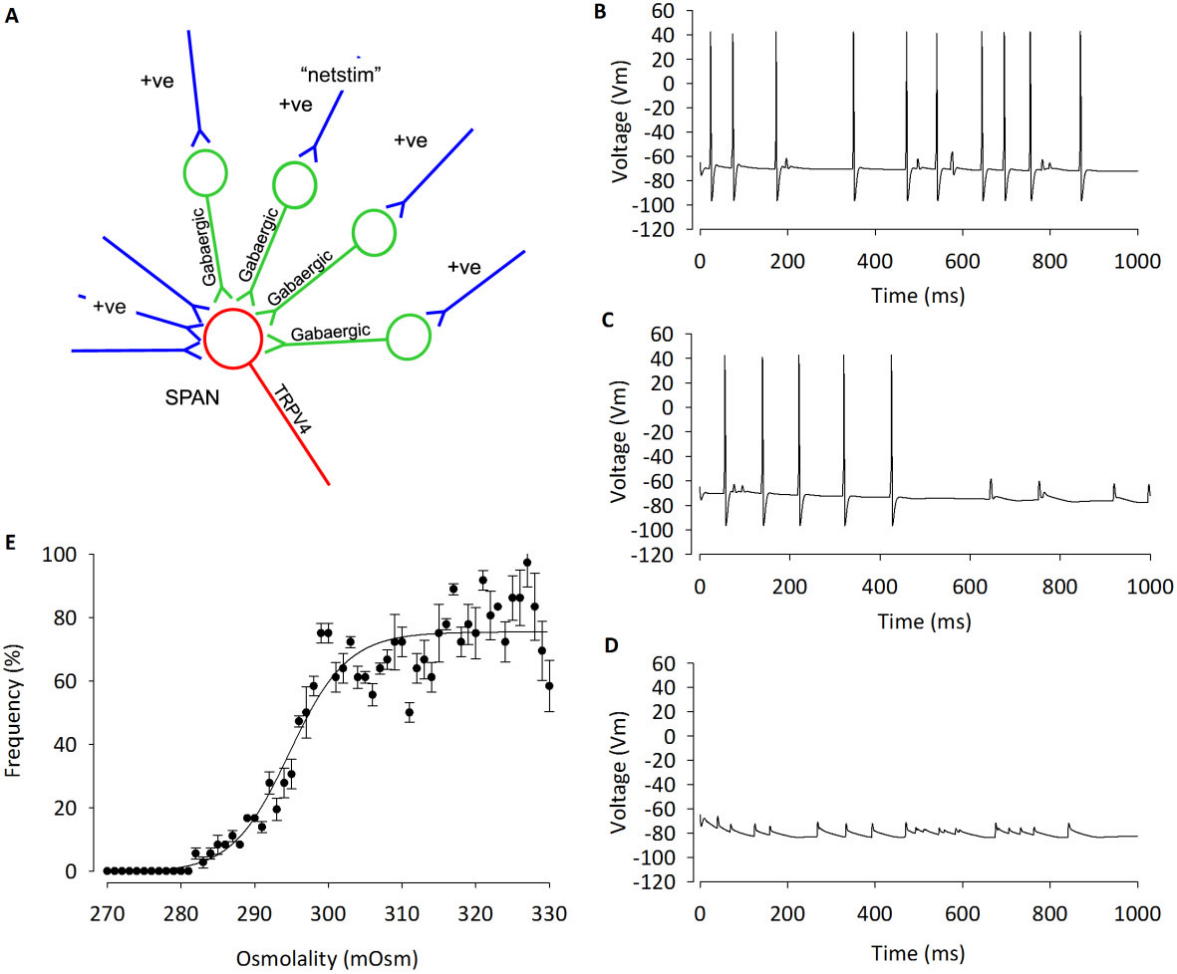
Development of a NEURON simulation based upon our hypothesis and parameters set by experimental data. (A) A computer model was developed using NEURON, which includes TRPV4 channels and allows an accumulation of Ca^2+^ into the cell. This is then linked to a K_Ca_ channel. Within the model, we can change osmolality and also block Ca^2+^ conductance. (B) Action potentials during hypertonic conditions (320 mOsm). (C) Decrease in action potential frequency when osmolality is reduced to 300 mOsm, and (D) further decreased action potential frequency at 270 mOsm. (E) Dose–response curve during changes in osmolality within the model.

**Figure 7:**
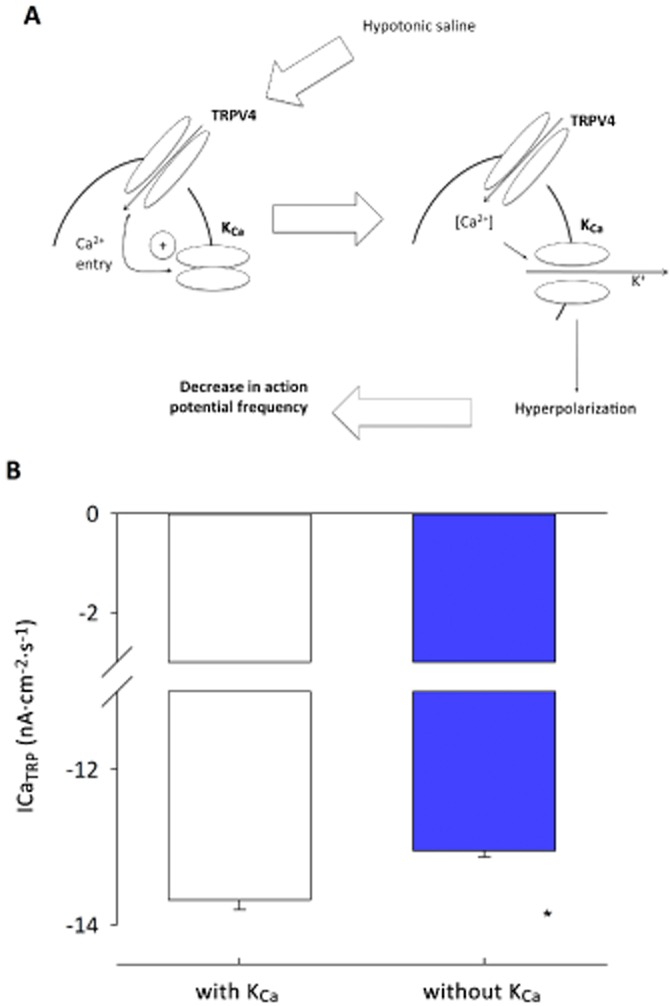
Analysis of positive feedback between TRPV4 and K_Ca_ channels in PVN neurones. (A) The simple scheme proposed by Nilius and Droogmans (2001) whereby influx of Ca^2+^ increases K_Ca_ channel activity which hyperpolarizes the cell and increases the inward flux of Ca^2+^, by increasing the driving force (*G* [*Vm* – *E*_Ca_]) for Ca^2+^ entry. (*Vm* = membrane potential, *E*_Ca_ is the equilibrium potential for Ca^2+^ ions and *G* is the conductance of the Ca^2+^ entry pathway). (B) Running a number of simulations reveals a significant decrease (*n* = 7; *P < 0.001) in mean Ca^2+^ influx in the presence of K_Ca_ channels, as predicted. See Figure 6 for experimental verification.

Our working hypothesis was that TRPV4channels, intracellular Ca^2+^ and K_Ca_ channels functionally couple in parvocellular PVN neurones, but it was not possible to test all these parameters simultaneously by experiment. Therefore, to verify our working hypothesis numerically, we extended our existing NEURON models to include TRPV4 and K_Ca_ channels, along with intracellular Ca^2+^ buffering (Carnevale and Hines, 2006). We used values derived from our *in vitro* experiments to model this system (Table 1). The model simulates the activation of TRPV4 channels by hypo-osmolality and closely matches our experimental data. Output from the model led to action potential frequency decreasing with decreases in osmolality (Figure 6B–E). The osmolality at which action potential frequency was half maximum was 297 ± 0.4 mOsm (Figure 6E). Furthermore, simulated block of either TRPV4 or K_Ca_ channels prevented this effect. We went on to test positive feedback, whereby K_Ca_-induced hyperpolarization leads to an increase in Ca^+2^ driving force and draws in further Ca^2+^ (Figure 7A). We simulated TRPV4 channel activity and calculated intracellular Ca^2+^, comparing intracellular Ca^2+^ with K_Ca_ channel conductance and with reduced conductance. Breakage of this positive feedback loop resulted in significantly reduced TRPV4 current and intracellular decrease of Ca^2+^ (Figure 7B; 12 nA·cm^2^·s^−1^ vs. 8 nA·cm^2^·s^−1^; *n* = 7; *P* < 0.001).

**Figure 8:**
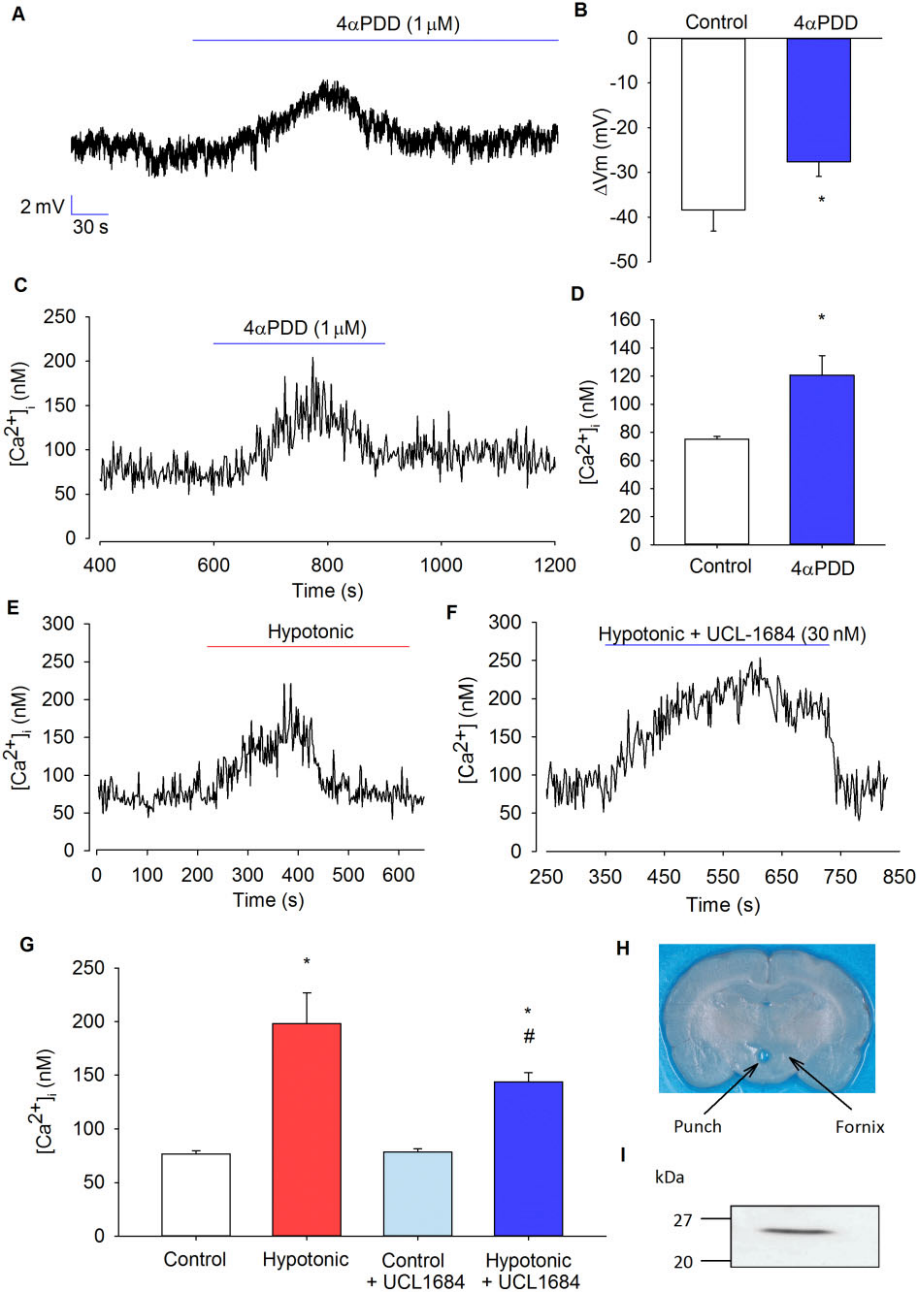
Whole-cell and Ca^2+^ in isolated PVN neurones. (A) Representative whole-cell current-clamp trace showing depolarization of the cell upon addition of 4αPDD. (B) Mean resting membrane potential of −54 ± 5 mV was recorded from several experiments as illustrated in (A), and a depolarization of 11 ± 2 mV was observed with 4αPDD (*n* = 4) *P < 0.05, signficantly different from control. (C) Representative Ca^2+^ trace showing a transient increase upon activation of TRPV4 channels in intact isolated neurones. (D) Mean intracellular Ca^2+^ from several experiments, as illustrated in (C), shows a significant transient increase in Ca^2+^ with 4αPDD (*n* = 6). *P < 0.005, signficantly different from control. (E) Representative Ca^2+^ trace showing a transient increase at 270 mOsm (hypotonic) in intact isolated neurones. (F) Representative Ca^2+^ trace showing a sustained increase in the presence of the SK channel inhibitor UCL-1684 at 270 mOsm in intact isolated neurones. (G) Mean intracellular Ca^2+^ from several experiments, as illustrated in (E), shows that Ca^2+^ levels are significantly increased at 270 mOsm (*n* = 10), with and without the presence of UCL-1684, compared with control (300 mOsm) (*n* = 13). *P < 0.001, signficantly different from control; *#P < 0.001, signficantly different from *control and #hypotonic). Ca^2+^ rise with hypotonic challenge was significantly reduced and sustained when cells are superfused with UCL-1684 (*P* < 0.05). (I) Western blot analyses of homogenates of cells from (H) tissue from PVN punch and immunoblotted with polyclonal antibodies against corticotropin-releasing factor (CRF). A strong immunoreactive band was detected at ∼25 kDa, consistent with the expression of CRF.

### Intracellular Ca^2+^ response to osmotic challenge

To investigate our model predictions of the effects of hypotonicity on intracellular Ca^2+^, we isolated PVN neurones with standard methods. We began by confirming membrane potential response to TRPV4 channel activation. Neurones exhibited a resting membrane potential of −54 ± 5 mV (*n* = 4) and were depolarized 11 ± 2 mV by 4αPDD superfusion (Figure 8A and B; *n* = 4; *P* < 0.05). Intracellular Ca^2+^ was measured with the ratiometric dye Fura-2AM. Addition of the TRPV4 channel activator, 4αPDD, significantly increased [Ca^2+^]_i_ (Figure 8C and D; from 75 ± 2 to 121 ± 14 nM, *n* = 6; *P* < 0.005). Hypotonic challenge also caused a transient increase in [Ca^2+^]_i_ (Figure 8F; from 77 ± 3 to 198 ± 28 nM, *n* = 8; *P* < 0.001).

Our model predicts that increase of [Ca^2+^]_i_ due to activation of TRPV4 channels will, in turn, activate K_Ca_ channels, creating a positive feedback loop by increasing the driving force for Ca*^2+^* entry. In order to verify experimentally whether the SK channel is involved in such a positive feedback circuit, we used the SK channel inhibitor UCL-1684 and applied a hypotonic challenge to the cells while measuring [Ca^2+^]_i_ with Fura-2AM. In the presence of UCL-1684 (30 nM), the increase in [Ca^2+^]_i_ was sustained but significantly reduced compared with the rise seen with hypotonic challenge alone (Figure 8F and G; from 121 ± 28 to 65 ± 8 nM, *n* = 13; *P* < 0.05). No difference was seen in basal [Ca^2+^]_i_ when cells were in the presence of UCL-1684 (*P* > 0.05).

## Discussion and conclusions

In this study, we report that central hypo-osmotic challenge reduces parvocellular neuronal activity and that the TRPV4 and, specifically SK channels are critical to this process. We provide the first direct evidence that TRPV4 channels functionally couple to SK channels to decrease parvocellular action potential frequency in response to hypo-osmolality.

### Parvocellular PVN neurones

Several lines of evidence demonstrate that the parvocellular subnucleus of the PVN includes neurones that modulate the cardiovascular system (Badoer, 2001; Coote, 2007; Pyner, 2009; Nunn *et al*., 2011). Knowledge of the pharmacology of PVN parvocellular neurones is limited, but in addition to amino acid neurotransmitters, adenosine and tachykinin receptors are known to be important (Womack and Barrett-Jolley, 2007; Culman *et al*., 2010; Li *et al*., 2010). In our own previous studies, we found substance P to act via disinhibition of GABAergic neurones (Womack *et al*., 2007). While several lines of evidence suggest that they may have a role in mediating pressor responses to psychological stress (Gabor and Leenen, 2011), other work shows that PVN neurones are important for variations in day–night sympathetic nerve activity (Feetham and Barrett-Jolley, 2014), thermoregulation, plasma volume control or osmosensing (Lovick *et al*., 1993; Eggermann *et al*., 2003; Stocker *et al*., 2004; Cham and Badoer, 2008; Haam *et al*., 2012). Under ordinary physiological circumstances, a number of nuclei in the hypothalamus contribute to the maintenance of plasma osmolality within a narrow range (∼290–300 mOsm). However, in extreme cases, for example, iatrogenically in hospitals (i.e. dialysis) or after MDMA use, osmolality can fall as low as 250 mOsm (Brvar *et al*., 2004), which results in dangerously low BP (Henrich *et al*., 1980). Several reports have shown that the supraoptic nucleus and the PVN are both important for sensing and responding to osmolality (Bourque, 2008), but in this study, we focused on PVN parvocellular neurones due to their established involvement with cardiovascular control (Coote, 2007).

### Functional coupling of TRPV4 and SK channels in parvocellular PVN neurones

The TRPV4 channel is a mechanosensitive ion channel known to be activated by osmolality changes and has a known role in cell volume control in other tissues (Liedtke and Friedman, 2003a; Becker *et al*., 2005; Phan *et al*., 2009; Guilak *et al*., 2010; Benfenati *et al*., 2011). Intravenous injection of the TRPV4 channel agonist 4αPDD decreases BP (Gao and Wang, 2010), and while some of this action may be of peripheral origin, it has been suggested that TRPV4 channels in the CNS may be responsible for volume control and osmosensing (Liedtke and Friedman, 2003a; Bourque, 2008). Our brain slice experiments confirmed that anatomically and morphologically defined parvocellular neurones responded to osmolality by showing that exposing the PVN to decreasing osmolality decreases ACf. This effect was mimicked by the TRPV4 channel agonists 4αPDD and GSK1016790A and reduced by its inhibitors RN1734 and HC067047. Interestingly, the difference in reduction of ACf between GSK1016790A and 4αPDD was significantly different; this could be due to the action of these agonists or a difference in specificity.

As these experiments were performed in slices, there is the potential for the effects observed to be indirect. In order to confirm the presence of TRPV4 channels on the parvocellular neurones patched, single-channel analysis was performed. A TRP-like channel was identified based upon conductance, amplitude and *V*_rev_. Upon the addition of the TRPV4 channel activator, 4αPDD, open probability of this channel increased significantly, confirming that it is indeed the TRPV4 channel.

We then went on to repeat brain slice protocols with similar experimental design to identify which K_Ca_ channel subtypes were involved in this process. Using available pharmacological inhibitors, it is possible to distinguish between SK, IK and BK channels. We used carefully selected concentrations of these inhibitors and found that the effect of hypotonicity on ACf was reversed by the selective SK channel inhibitor UCL-1684, but not by selective inhibitors of BK or IK channels. This effect may be somewhat surprising, as BK channels have been found in the parvocellular PVN previously, and their inhibition changes action potential duration and frequency (Salzmann *et al*., 2010). There are at least two possibilities as to why there is no effect on action potential frequency when IbTX was used: (i) simply, the BK channels in these cells are not in close enough proximity to the TRPV4 channels to be affected by localized Ca*^2+^* entry and so are not involved in this process, or (ii) as BK channel activation is highly dependent upon voltage; cell depolarization resulting from an influx of Ca^2+^ could mean that the BK channels would remain active regardless of the introduction of IbTX (Contreras *et al*., 2012).

Furthermore, a decrease in ACf was observed when the TRPV4 channel activator GSK1016790A was applied alone to parvocellular neurons in slices but, in the presence of the SK channel inhibitor, UCL-1684, this TRPV4 channel activator was without effect on ACf. Presumably, there was still an influx of Ca^2+^ due to activation of the TRPV4 channel, but the SK channel was not activated by the rise in intracellular Ca^2+^ in this instance due to the block imposed by UCL-1684. This provides clear evidence to suggest that these two channels are functionally coupled.

### *In silico* model of osmotic control of parvocellular PVN neurones

Several lines of evidence show that TRPV4 channels are present within the parvocellular region of the PVN and is involved in osmosensing in the PVN. We investigated this potential role in our existing working model of spinally projecting neurones.

Studies in several different tissues have suggested that influx of Ca^2+^ via TRPV4 channels would lead to the activation of one or more of the K_Ca_ channels locally (Arnhold *et al*., 2007; Gao and Wang, 2010). This simple hypothesis has been widely discussed. Furthermore, Nilius and Droogman proposed that in some cells (endothelial in particular), activation of K_Ca_ channels would cause hyperpolarization, which, in turn, would draw greater Ca^2+^ into the cell by increasing the driving force for Ca^2+^ entry (*Vm-E*_Ca_, where *E*_Ca_ is the equilibrium potential for Ca^2+^ ions and *Vm* is the membrane potential), setting up a positive feedback loop (Nilius and Droogmans, 2001; Watanabe *et al*., 2002). We felt that it was important to investigate whether these simple ideas would stand up to numerical simulation. There are a number of ways to approach this, but we used NEURON as it is widely validated and has several readily available sub-routines to simulate such factors as K_Ca_ channels, Ca^2+^ accumulation and Ca^2+^ pumps. To this end, we created a computer simulation based upon our standard model of a PVN parvocellular neurone (Lewis *et al*., 2010), but now extending this to include a TRPV4 channel conductance, K_Ca_ channel conductance and NEURON Ca^2+^ buffering routine. This model (Figure 6A) assumes a tonic excitatory input together with considerable basal inhibition by GABAergic inputs, as this has been shown both *in vitro* (Barrett-Jolley, 2001; Zaki and Barrett-Jolley, 2002; Park *et al*., 2009) and *in vivo* (Martin *et al*., 1991; Zhang and Patel, 1998). The numerical model then simulates activation of TRPV4 channels with decreasing osmolality, leading to an accumulation of Ca^2+^ within the cell (Figure 7A), as have gone on to verify experimentally. The model confirms that sufficient K_Ca_ channels would be activated to reduce firing of action potentials via an evoked hyperpolarization. The model predicts that decreasing osmolality will reduce action potential frequency, a result that we see reliably *in vitro*. The model confirms that the Nilius–Droogman positive feedback effect is also possible in PVN neurones, which again we have confirmed *in vitro*.

### Experimental verification of the coupling of TRPV4 and SK channels in parvocellular PVN neurones

Although the experiments using slices are more physiological, due to the complex nature of the tissue, it is difficult to ascertain if the environment change/drug intervention affects the cell we are recording from directly, or as a result of some other upstream effect. For this reason, we moved on to isolated cells to investigate the effects of activating TRPV4 channels on the whole cell and intracellular calcium concentrations to test our model. Unlike the slices, these cells are quiescent in nature due to their lack of innervation. We showed that the TRPV4 channel agonist, 4αPDD, depolarized these neurones during whole-cell recordings and that there was a transient increase in Ca^2+^, confirming the presence of a negative feedback circuit in this process. This depolarization may seem counter-intuitive as our model predicts that activation of TRPV4 channels would ultimately lead to a hyperpolarization of the cell via activation of K_Ca_ channels (SK), a hypothesis that was supported by reduced action current firing with activation of TRPV4 channels. We therefore suggest that the observed depolarization could be as a result of the necessary presence of a calcium chelator in the whole-cell intracellular pipette solution. In our whole-cell experiments, we used EGTA which appeared to have prevented Ca^2+^ to rise high enough to allow subsequent activation of SK channels.

We have shown, during Ca^2+^ measurements, that hypotonic challenge also results in a transient increase in Ca^2+^. Using the specific SK channel inhibitor UCL-1684, a sustained increase in Ca^2+^ was seen, possibly due to insufficient levels of intracellular Ca^2+^ to trigger negative feedback. We hypothesize that this is due to the inhibition of the SK channel, and so a decrease in the driving force for Ca^2+^ entry into the cell (i.e positive feedback is first prevented), further pointing towards a coupling between the TRPV4 and SK channels. These feedback systems introduce some time-dependency to this mechanism, as is evident from whole-cell and Ca^2+^ measurements. We took this into account with the inclusion of forward and reverse rate parameters in our mathematical model. Interestingly, with action current osmolality experiments, this is not always seen, with the treatment having a prolonged effect. This is perhaps due to these recordings being performed in slices, with the presence of additional contributory mechanisms involved with osmosensing. Our working model of this system also generates a number of predictions that could be tested in future experiments. For example, as discussed earlier, in Figure 8 we show that under conventional whole-cell patch (current clamp) configuration, 4αPDD induces a small depolarization. Our model suggests that in intact cells, this would result in hyperpolarization as Ca^2+^ rises and SK channels are opened. Experiments employing reduced pipette EGTA, perforated patch or indeed voltage sensitive dyes may provide further insight. Furthermore, as we have concluded that the TRPV4 channels are the major component underlying the intracellular Ca^2+^ elevation, we would predict that this Ca^2+^ rise and associated hyperpolarization would be abolished by removal of extracellular Ca^2+^ or indeed, TRPV4 channel antagonists (Alexander *et al*., 2011), siRNA or appropriately targeted monoclonal antibodies (Benham, 2005).

Our data show for the first time that coupling of TRPV4/SK channels is essential for osmosensing by PVN neurons. Although this osmosensitive apparatus could be located on either PVN input neurones or the parvocellular PVN neurones themselves, as it is known that parvocellular neurones express both TRPV4 and SK channels themselves, the simplest hypothesis would be that the location of the ‘osmosensor’ is the parvocellular neurone. This is further supported by our single channel recordings in parvocellular neurons and experiments in isolated neurones which confirm a direct role for osmosensing in the PVN. Our experimental data therefore lead us to the conclusion that central TRPV4 channels play a role in osmosensing within the parvocellular PVN. While recent work confirms that the PVN is important for coupling osmolality to sympathetic nerve activity *in vivo* (Holbein *et al*., 2014), further experiments will be required to investigate if these involve TRPV4 and SK channel coupling. Interestingly, it was recently shown that thirst itself does not appear to involve TRPV4 channels as TRPV4−/− mice had no change in *ad libitum* water consumption (Kinsman *et al*., 2014).

In conclusion, our data show that hypotonic challenge activates the mechanosensitive TRPV4 channel and therefore increases intracellular Ca^2+^. This accumulation of Ca^2+^ within the cell appears to activate SK channels, hyperpolarizes neurones and thence decreases action potential firing frequency. Furthermore, our data provide evidence of both positive and negative feedback processes within this mechanism. As the neurones within the parvocellular PVN are known to have sympathetic influence, further experiments would be required to establish if this coupling mechanism underlies central osmolality detection *in vivo*.

## Abbreviations

4αPDD: 4-α-phorbol 12,13-didecanoate
ACf: action current frequency
ACSF: artificial cerebro-spinal fluid
BK: large-conductance calcium-activated potassium channel
IK: intermediate-conductance calcium-activated potassium channel
K_Ca_: calcium-activated potassium channel
PVN: paraventricular nucleus
*R*: ratio
*R*_max_: maximum ratio
*R*_min_: minimum ratio
SK: small-conductance calcium-activated potassium channel
TRPV4: transient receptor potential vanilloid channel 4

## Supporting Information

**Figure S1** Kinetic analysis of TRP-like channels. (A) Example of raw dated fitted with an idealised trace. in the absence (control) and presence of 4αPDD (1 μM) 4αPDD. Rate constants were fitted to the idealized record using the simple model illustrated in (B). **Table S1** Rate constants for the TRP-like channel with and without the presence of 4αPDD (1 μM) and fitted to the idealized record using the model in Supporting Figure S1B. **P* < 0.005.
