# Supplementary material for: TRPV4 and KCa ion channels functionally couple as osmosensors in the paraventricular nucleus
Source: Br J Pharmacol. 2015 Jan 23;172(7):1753–68. doi: 10.1111/bph.13023 (PMC4376454; doi:10.1111/bph.13023)
Supplement: Supplementary file 1 [file bph0172-1753-sd1.pdf]

Supporting information

Single channel kinetics

Cell-attached patch recording conditions were identical to those described in the main paper, and similar to those described (Lewis *et al.*, 2013). Following activation of ion channels by 4αPDD it was apparent that most patches had more than one ion channel present. Using a the model based event-detection, segmental K means (SKM) methods of Qin (Qin, 2004) (with QuB software, Dr. Feng Qin, Dr. Lorin Milescu, Fu Qiong, Chris Nicolai, and John Bannen, SUNY, Buffalo, NY) record idealisation is still possible, however, it weakens the confidence of kinetic analysis (decreases log-likelihood) and decreases overall signal to noise ratio. To address this we digitally re-filtered data to 250Hz. Models were fit to the idealized record by optimisation of maximum interval likelihood (Qin *et al.*, 1996; Qin *et al.*, 1997). We chose the simplest model (Supporting Figure 1) which could be used to fit all 4 of the patches analysed in both the presence and absence of drug.

References

Lewis R, Feetham CH, Gentles L, Penny J, Tregilgas L, Tohami W, *et al.* (2013). Benzamil sensitive ion channels contribute to volume regulation in canine chondrocytes. *Br J Pharmacol* **168**(7)**:** 1584-1596.

Qin F (2004). Restoration of single-channel currents using the segmental k-means method based on hidden Markov modeling. *Biophys J* **86**(3)**:** 1488-1501.

Qin F, Auerbach A, Sachs F (1996). Estimating single-channel kinetic parameters from idealized patch-clamp data containing missed events. *Biophys J* **70**(1)**:** 264-280.

Qin F, Auerbach A, Sachs F (1997). Maximum likelihood estimation of aggregated Markov processes. *Proceedings of the Royal Society of London Series B-Biological Sciences* **264**(1380)**:** 375-383.

Supporting Information Figure 1

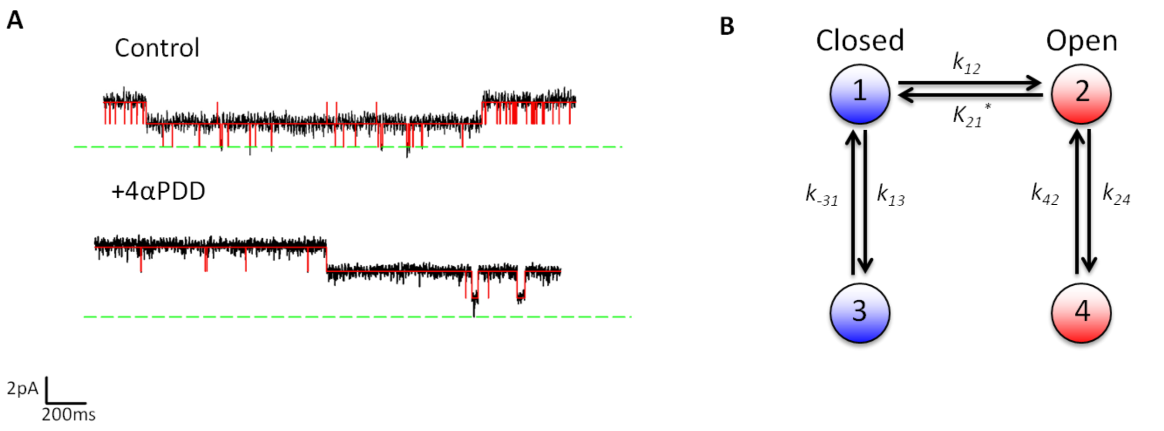

Supplementary Figure 1: Kinetic analysis of TRP-like channels. (A) Example of raw dated fitted with an idealised trace. in the absence (control) and presence of 4αPDD (1 µM) 4αPDD. Rate constants were fitted to the idealized record using the simple model illustrated in (B).

Supplementary Table 1: rate constants for the TRP-like channel with and without the presence of 4αPDD (1 µM) and fitted to the idealized record using the model in Supporting Figure 1 (B) *p<0.005.

	**Control (s^{-1})**	**+4αPDD (s^{-1})**
k_{12}	235±34 (n=4)	326±35 (n=4)
k_{21}	255±62 (n=4)	61±27 (n=4) *
k_{24}	64±13 (n=4)	29±17 (n=4)
k_{42}	15±6 (n=4)	28±13 (n=4)
k_{13}	33±5 (n=4)	49±17 (n=4)
k_{31}	10±2 (n=4)	5±2 (n=4)
